# Biomarker- and similarity coefficient-based approaches to bacterial mixture characterization using matrix-assisted laser desorption ionization time-of-flight mass spectrometry (MALDI-TOF MS)

**DOI:** 10.1038/srep15834

**Published:** 2015-11-05

**Authors:** Lin Zhang, Sonja Smart, Todd R Sandrin

**Affiliations:** 1 School of Mathematical and Natural Sciences, Arizona State University, Phoenix, AZ 85069

## Abstract

MALDI-TOF MS profiling has been shown to be a rapid and reliable method to characterize pure cultures of bacteria. Currently, there is keen interest in using this technique to identify bacteria in mixtures. Promising results have been reported with two- or three-isolate model systems using biomarker-based approaches. In this work, we applied MALDI-TOF MS-based methods to a more complex model mixture containing six bacteria. We employed: 1) a biomarker-based approach that has previously been shown to be useful in identification of individual bacteria in pure cultures and simple mixtures and 2) a similarity coefficient-based approach that is routinely and nearly exclusively applied to identification of individual bacteria in pure cultures. Both strategies were developed and evaluated using blind-coded mixtures. With regard to the biomarker-based approach, results showed that most peaks in mixture spectra could be assigned to those found in spectra of each component bacterium; however, peaks shared by two isolates as well as peaks that could not be assigned to any individual component isolate were observed. For two-isolate blind-coded samples, bacteria were correctly identified using both similarity coefficient- and biomarker-based strategies, while for blind-coded samples containing more than two isolates, bacteria were more effectively identified using a biomarker-based strategy.

Matrix-assisted laser desorption/ionization time-of-flight (MALDI-TOF) mass spectrometry (MS) has been shown to facilitate rapid and accurate identification of bacteria isolated in clinical labs, food processing, and many diverse environments. Reliable characterization at the genus, species, and in some cases, strain levels has been reported^1^. Peaks of biological molecules, typically proteins, which originate from cell surfaces, intracellular membranes and ribosomes, constitute fingerprints of the bacterium analyzed^2^. These unique fingerprints (mass spectra) are typically compared with spectra in databases by two approaches, biomarker- and similarity coefficient -based, for identification. In general, biomarkers are peaks identified in spectra, whose presence indicates the presence of certain species or strains. Similarity coefficient-based approaches measure the degree to which spectra (unknown vs. reference) are alike. One of the most commonly used similarity coefficients is the Pearson correlation coefficient^2^.

MALDI-TOF MS profiling has been most commonly used to characterize pure cultures. Accordingly, bacteria must be isolated in pure culture prior to analysis^3^. Isolation and cultivation are time-consuming and may cause biased results. For example, isolation significantly lengthens the time required to complete diagnostic procedures in clinical labs. With regard to environmental bacteria, slow-growing environmental isolates may need several days to form colonies on agar plates^4^,^5^,^6^. Furthermore, isolation techniques and cultivation media have been shown to affect MALDI-based bacterial differentiation, especially at the strain level^7^,^8^. As a result, there has been considerable interest in applying rapid MALDI-based techniques to characterize samples without pure culture isolation. Successes using direct characterization without pure culture isolation have been described with monomicrobial-contaminated blood samples, urine samples, milk, and plant tissues^4^,^9^,^10^,^11^,^12^,^13^,^14^,^15^,^16^,^17^.

Studies have also investigated use of MALDI to characterize simple mixtures (polymicrobial samples) without pure culture isolation^18^,^19^,^20^,^21^. These simple mixtures are model systems usually constructed by mixing equal amounts of two or three bacteria^18^,^19^,^20^,^21^. Component bacteria in these model systems have been identified using a manner similar to a biomarker-based method, but those efforts have been limited. Specifically, only one biomarker peak of each component species was observed in the spectra of the mixtures when manually comparing the mixture spectra with the reference spectra of pure cultures^9^,^22^. In addition to model systems, clinical samples such as positive blood cultures and urine samples have also been studied. These clinical specimens usually contained two bacterial species occurring in varied ratios. Failure of identification of one or two species has been reported^23^,^24^, possibly due to the unequal representation of the two species in the mixture. It has been suggested that the bacterium in the mixture occurring at a lower concentration can be detected by MALDI only when its concentration is higher than 5% of the mixture^18^. These results suggest that uses of MALDI-TOF MS to directly characterize polymicrobial samples are more challenging than those involving pure culture isolations and monomicrobial samples. Recently, Mahe *et al.* successfully identified bacterial components using model systems containing two species. The identification procedure was to step-by-step subtract the individual peak profiles from mixture spectra and then compare remaining peaks to the routine databases, which used a biomarker-based approach^21^. This indicates that a routine database may be applicable for characterization of mixtures with appropriate characterization methods. To further explore the feasibility of using MALDI to characterize mixed samples, more complex model systems containing a broader ranges of species need to be investigated, as these model systems may better represent diversity commonly found in environmental samples. Furthermore, similarity-coefficient based methods need to be thoroughly evaluated as they are routinely and effectively used for MALDI-based characterization of individual bacteria.

Here, we report using MALDI-TOF MS to characterize a more complex model mixture containing six environmental bacteria isolated from a unique cave environment (Kartchner Caverns, AZ, USA). Environmental isolates were chosen instead of more well-characterized, medically-relevant isolates to explore and develop strategies that might expand the utility of MALDI-based microbial fingerprinting to more diverse, less well-characterized mixtures of microorganisms. Each of these bacteria have been rigorously characterized individually using MALDI fingerprint-based methods previously^25^. The model mixture was constructed by mixing equal volumes of broth pure cultures with equal optical densities. Mass spectra of inactivated protein extracts were acquired for both pure cultures and the model mixture. Blind-coded mixture samples were constructed and tested for identification of bacteria from polymicrobial samples. Results suggest that MALDI-TOF MS fingerprint-based methods can be applied to identify component isolates based on mixture spectra and a database of isolates.

## Results

### Mass spectra

**Figure 1:**
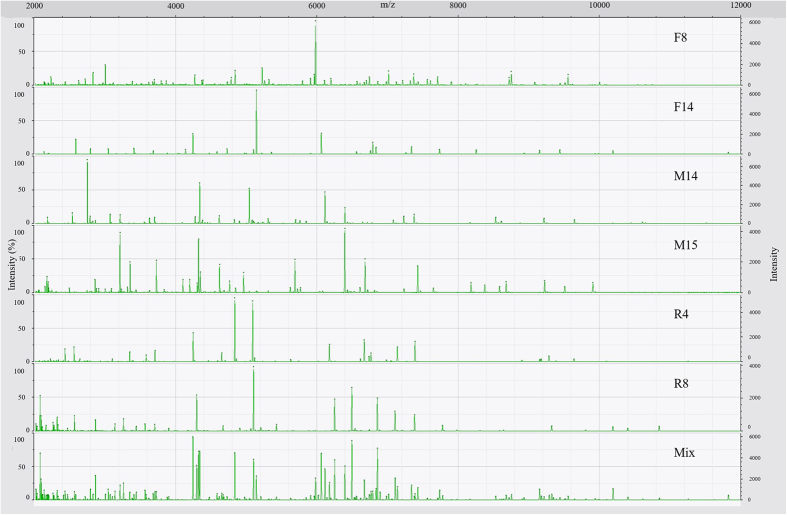
Representative mass spectra of six environmental isolates (F8, F14, M14, M15, R4, and R8) and the model mixture system (Mix).

As expected, spectra of the model mixture were more complex than spectra of the individual isolates that composed the mixture (Fig. 1; Table 1). More peaks were observed in the spectrum of the mixture than in the spectra of pure cultures. The spectrum of the mixture contained 135 ± 10 peaks, while the spectra of the pure cultures contained numbers of peaks ranging from 29 ± 3 for F14 to 63 ± 6 for F8 (Table 1). With regard to the mass range, peaks in the spectrum of the model mixture ranged from 2,026 to 11,825 Da. This covered the entire mass ranges of the spectra of the six individual isolates, which were from 2,026 Da (the lowest mass observed in the spectra of isolates) for R8 to 11,822 Da (the highest mass observed in the spectra of isolates) for F14 (Table 1). All spectra had high reproducibility, ranging from 96.0 ± 2.4% for F8 to 99.7 ± 0.2% for F14 (Table 1).

### Isolate representation in mixture mass spectra

Peaks in the replicate spectra of the model mixture were matched to the peaks in the spectra of the six isolates (Table 2). Only peaks observed in all three replicates of each isolate were considered for matching. Some peaks in the spectra of the model mixture were shared by two isolates. For example, m/z 3,709 was shared by R4 and R8, and m/z 6,673 was shared by F8 and R4 (Supplementary Table 1). No peaks were shared by more than two isolates. Shared peaks were counted for both isolates. The isolate R8 had highest number of peaks represented in the spectra of the model mixture, while M14 had the least (Table 1). Specifically, the order was R8 (35 peaks) > F8 (26 peaks) > F14 (22 peaks) > R4 (20 peaks) > M15 (14 peaks) > M14 (13 peaks) (Table 2).

Considering that the spectra of pure cultures of each isolate contained different numbers of peaks in the mixture spectrum, a percentage of presence (PP) was calculated for each isolate to further quantify representation of each isolate in the spectrum of the mixture (Table 2). Specifically, Eq. 1 was used to calculate PP for each isolate by dividing the number of peaks associated with that isolate that were observed in the spectrum of the mixture (N_m_) by the number of peaks in the spectrum of the pure culture of that isolate (N_p_):


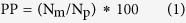


Nearly 90% of R8 peaks were present in the spectrum of the model mixture, while only 23.3% of M15 peaks were present in spectra of the model mixture. Specifically, the order is R8 (89.7%) > F14 (75.9%) > R4 (55.6%) > F8 (41.3%) > M14 (34.2%) > M15 (23.3%).

Interestingly, 13 peaks present in the spectrum of the model mixture did not belong to any of the individual isolates, among which five peaks were not observed in any replicate spectrum of the pure cultures, while the other 8 peaks were shown in 1 or 2 replicate spectra of the pure cultures (Supplementary Table 2). All of these “extra” peaks had intensities higher than 100 a.u. and lower than 500 a.u. except one peak m/z 6,897 (Supplementary Table 2), which reached 751 a.u (Supplementary Table 2).

**Figure 2:**
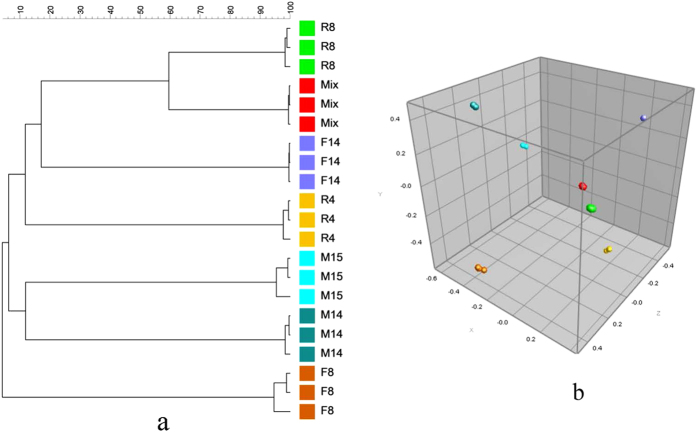
Cluster analysis (**a**) and multidimensional scaling (MDS) (**b**) for spectra of six environmental isolates and the model mixture system.

Cluster analysis based on curve-based Pearson correlation coefficients suggested that the replicate spectra of the model mixture were more similar to the replicate spectra of R8 and F14 than to the spectra of other isolates (Fig. 2a). This was also apparent using multidimensional scaling (MDS) analysis (Fig. 2b).

### *In silico* synthesis of mixture mass spectra using mass spectra of pure cultures

A synthetic mixture spectrum (SMS) of the six-isolate model mixture was generated *in silico* using the spectra of pure cultures in the database to incorporate all 18 replicate spectra of the 6 isolates into a single spectrum. Peak positions were created in the SMS as described previously^25^ using position tolerance values that were calculated as follows:


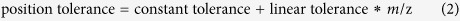


Constant tolerance equaled 1.9 and linear tolerance equaled 550^25^. Furthermore, a peak was only included in the SMS if more than 16% (3 out of 18 spectra) of the spectra exhibited peaks at the position. The intensity of each peak in SMS was reported as the averaged value of the intensities of peaks in the individual spectra of pure cultures.

**Figure 3:**
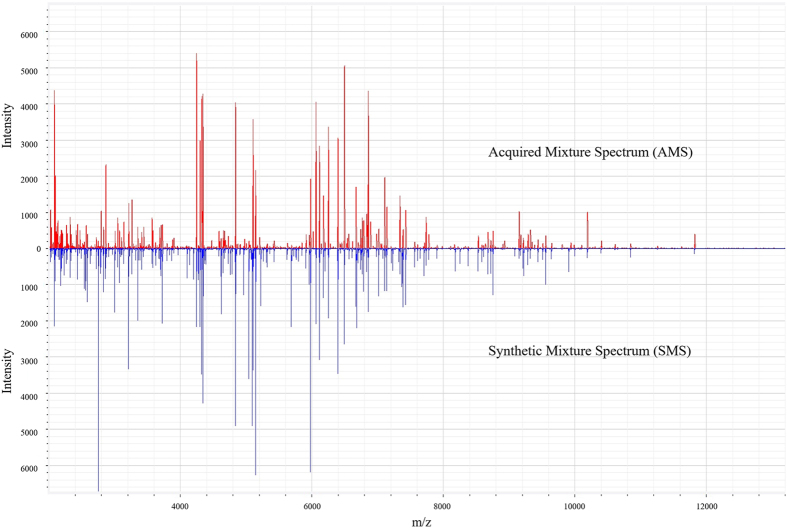
A comparison of spectra of the model mixture (acquired mixture spectrum) and the summarized spectra using spectra of pure cultures (synthetic mixture spectrum).

The SMS and the corresponding mixture spectrum acquired using the six-isolate model mixture (designated as the acquired mixture spectrum, AMS, appeared similar (Fig. 3). The AMS contained 145 peaks, while the SMS contained 195 peaks, indicating that some peaks of component isolates were not detectable when characterizing a mixture of them. The similarity between these two spectra was 68.6%. When preliminarily applying smoothing to spectra, results showed that with 0.5% smoothing, the similarity (Pearson correlation coefficient) between the AMS and SMS increased from 68.6% to 75.7%, and the similarity further increased to 79.6% with 1% smoothing. This suggests that minor smoothing of spectra may affect the similarity between AMS and SMS. Investigation regarding such spectrum processing parameters may be needed for further optimization of complex mixture analysis using SMS-based similarity coefficient methods.

### Identification of blind-coded mixtures

Blind-coded model mixtures were constructed by mixing equal volumes of each cell suspension in a single microcentrifuge tube (Table 3). Spectra of blind-coded mixtures were acquired using the same methods that were used for pure cultures and the six-isolate model mixture system. Before analyzing spectra of the blind-coded samples, isolate-specific peaks (potential biomarkers) were selected based on the peak-matching results, and only peaks with intensities higher than 500 a.u. were considered. In addition, SMS of blind-coded samples were constructed by summarizing the spectra of pure cultures using the composition of blind-coded samples.

For identification of the blind-coded mixtures, two strategies were applied: similarity coefficient-based and biomarker-based. The similarity coefficient-based strategy compared the replicate AMS of a blind-coded sample with the SMS of all blind-coded samples. The similarity coefficient was calculated using the Pearson correlation coefficient with 0% smoothing. An identification of the constituent species in each mixture was made when the similarity coefficient between AMS and SMS exceeded 68.6%. If no similarity coefficients reached 68.6%, 0.5% to 1% smoothing was applied. Table 3 shows the identification results using the similarity coefficient-based strategy. High similarity (~90%) was achieved for sample A and B, which each contained two isolates, while the similarity coefficient for sample C which also contained two isolates did not reach 68.6% even with 1% smoothing. Interestingly, using the 68.6% threshold value, multiple identification results were reported for samples E and F, which contained four and five isolates, respectively; however, correct identification was still achieved for these two samples with the highest similarity coefficient in the corresponding group of multiple results (Table 3).

A biomarker-based strategy was used to manually identify biomarkers for each isolate in the AMS of blind-coded samples (Table 4). Initially, to postulate the existence of an isolate in the sample, at least one potential biomarker peak of the isolate needed to be found in the spectra of the blind-coded sample. Further optimization was used to remove the isolate with only one biomarker peak shown from the initial identification results, since “shared peaks” were observed in mixture spectra (Supplementary Table 2). In contrast to the identification made using similarity coefficients, sample C was correctly identified using potential biomarkers; however, M15 in sample D could not be correctly identified because no potential biomarker peaks higher than 500 a.u. were observed in spectra of the mixtures.

## Discussion

In this study, MALDI-TOF mass spectra of a model mixture consisting of six environmental isolates were acquired and compared with mass spectra of each isolate composing the mixture. Results indicate that the mixture spectra are more complex than spectra of pure cultures in terms of number of peaks and mass range (Table 1). For example, mixture spectra contained 2 to 4 times more peaks than spectra of pure cultures, and the mass range of the mixture spectra covered the entire mass ranges of spectra of pure cultures. Most peaks in the mixture spectra could be assigned to each individual bacterium, but both shared and extra (mixture-specific) peaks were also present in the mixture spectra. “Shared” peaks are those that could be assigned to two isolates. The appearance of “shared” peaks has been reported previously, in which a more simple model mixture containing only two isolates was used^26^. Interestingly, though our model mixture was more complex than the two-isolate model system, there was no peak shared by more than two isolates. Mixture-specific peaks were those that could not be assigned to any individual bacterium. This rather curious phenomenon has also been observed previously when using a two-isolate model mixture, in which two mixture-specific peaks were observed^26^. In our six-isolate model mixture, more mixture-specific peaks were observed. Some were observed in only one or two replicate spectra, indicating that such peaks may result from bacteria cell extract components with inconsistent presence; however, five mixture-specific peaks in the spectra of the six-isolate model mixture were not observed in any replicate spectra of the pure cultures (Supplementary Table 2). These five peaks may be generated by reactions catalyzed by enzymes which were released from cells when extracting proteins. We hypothesize that complex samples which contain more than six bacterial isolates may contain even more mixture-specific peaks. We are further exploring whether the pattern and/or appearance of mixture-specific peaks can be used as representative biomarkers for bacterial mixtures.

Prior work has shown that varying amounts of individual bacteria in mixtures affects the representation of component bacteria in mixture spectra^18^. For this reason, we chose to use a single concentration of each bacterium to be able to directly and rigorously compare two different approaches to mixture characterization. Though the six-isolate model mixture was constructed using equal O.D. for each isolate, the numbers of peaks observed and the percentage of peak presence (PP) in the mixture spectra were different for each isolate. This indicates that some isolates may be disproportionately represented when profiling mixture samples using MALDI, as has been suggested previously^26^. This may be due to several reasons. For example, although the O.D. was adjusted to be equal, the cell number may have varied, resulting in an unequal concentration of released proteins for each isolate. The protein extraction protocol may yield more proteins for some isolates (e.g. Gram negative species) than others (e.g. Gram positive species). All of these factors may contribute to the reduced representation of a particular isolate in a mixture spectrum.

Cluster analysis showed that, generally, the profiles of mixture samples were more similar to the isolate which has higher percentage of peak presence in the mixture spectra than isolates with lower values of this metric. In contrast, the similarity trend was not consistent with the order of number of peaks represented in the mixture spectra. This is reasonable, because higher percentage of peak presence suggests more information about the isolate is contained in the mixture mass profiles. This indicates that, though the exact bacterial composition in mixture samples cannot be elucidated only by cluster analysis of mixture spectra and spectra of pure cultures, the species with mass profiles showing the highest similarity to those of mixture samples are very likely members of the mixture samples. Accordingly, further work to explore this technique as a screening method for rapid detection of predominant species in mixture samples is warranted.

With regard to identifying bacteria in mixtures, some previous studies have shown that species-specific peaks (potential biomarkers) of component bacteria can be observed in mixture spectra when using simple mixture model systems. The component bacteria can be identified based on identification of biomarker peaks, and usually, only one biomarker peak is investigated. In contrast, we observed shared peaks when using a considerably more complex model mixture. This suggests that the identification of bacteria from mass spectra of mixtures should not rely on a single biomarker peak of the isolates of interest. Sophisticated algorithms which examined the whole mass profiles of bacteria have been developed to automatically identify bacteria from mixture model systems^21^. Though effective, these algorithms are complex and not used routinely in clinical and environmental microbiology labs.

In summary, we employed two strategies, similarity coefficient- and biomarker-based strategies, to identify bacteria using mixture mass spectra and a database containing spectra of pure cultures. Since our model mixture was constructed using an equal OD for each component species, we hypothesized that mixture spectra may be generated by *in silico* synthesis of spectra of pure cultures. The SMS of the six-isolate model mixture showed 68.6% similarity to the AMS, and preliminarily data processing using a common smoothing algorithm further increased the similarity coefficients. Smoothing removes noise peaks in the spectra. Thus, the fact that smoothing increased the similarity between the SMS and the AMS indicates that low intensity peaks of each species may contribute less than high intensity peaks to the mass profiles of mixture samples. By comparing the AMS of blind-coded samples with various SMS, generally, high similarity was observed for samples containing two species. For samples containing more than two species, multiple results were observed, but the highest similarity coefficient in these multiple results reflected the correct composition of the mixture.

With regard to the biomarker-based strategy, our results showed that with limited numbers of biomarker peaks, for example, only one or two biomarker peaks, misidentification may occur. This highlights the need to further examine the threshold number of peaks to be examined when using biomarker-based strategies to identify individual bacteria in mixtures. Furthermore, these two strategies may perform differently with the same sample. For example, sample C could not be identified using the similarity coefficient-based strategy, while correct identification of constituent bacteria was obtained using the biomarker-based strategy. In contrast, only the similarity-coefficient-based strategy facilitated reliable identification of members of the mixture in sample D.

Overall, our results suggest that MALDI-TOF MS fingerprint-based methods have promise to identify bacteria in complex mixtures using mixture spectra and a database containing spectra of pure cultures. While neither approach imposed additional computational costs (i.e., calculation of relevant similarity coefficients and construction of SMS were nearly instantaneous in the software we employed), both identification strategies may need to be examined and optimized prior to application to particular mixtures to maximize performance. Accordingly, investigation of additional mixtures from other environments and of non-model mixtures in which variability is inherently higher is needed to further elucidate and optimize the use of this technique to rapidly characterize complex bacterial mixtures. In particular, polymicrobial blood cultures that pose challenges for existing workflows and products (e.g., Bruker’s Sepsityper) may be more readily, rapidly, and reliably characterized using such optimized strategies.

## Materials and Methods

### Reagents

Acetonitrile (≥99.7%) was purchased from Alfa Aesar (Ward Hill, MA, USA). Trifluoroacetic acid (≥99.5%) and α-cyano-4-hydroxycinnamic acid (CHCA) (≥97%) were purchased from ACROS (Fair Lawn, NJ, USA). Formic acid (≥88.0%) and MALDI calibrants were purchased from Sigma-Aldrich (St. Louis, MO, USA). Absolute ethanol was purchased from Thermo Fisher Scientific (Waltham, MS, USA). R2A agar was purchased from Carolina Biological Supply Company (Burlington, NC, USA).

### Bacteria, media, and construction of the model mixture

Bacteria used in this study were isolated from Kartchner Caverns, AZ, USA and stored as freezer stocks (50:50 R_2_B bacterial culture:glycerol) at −80 °C (Table 1). All six bacteria were identified using 16S rRNA gene sequencing to the genus level^25^. R_2_A plates were streaked from freezer stocks and incubated at room temperature for 2–3 days. A single colony from an R_2_A plate was used to inoculate R_2_B broth. All R_2_B broth cultures were incubated at room temperature on an orbital shaker at 200 rpm for 40 h. After incubation, the optical density of each broth was adjusted to 1.0 at 600 nm. A model mixture containing all six bacteria was made by mixing equal volumes of each cell suspension (O.D._600_ = 1) into a single sterile microcentrifuge tube.

### Sample preparation

All cell suspensions (O.D._600_ = 1) and the model mixture system were prepared for MALDI analysis using a protein extraction sample preparation method as previously described^27^. Briefly, for each sample, 1 mL (O.D._600_ = 1) was centrifuged at 17,000 × g for 5 minutes, and the supernatant was decanted. The cell pellet was inactivated by resuspension and incubation for 1 h in 300 μl of sterile dd H_2_O and 900 μl of absolute ethanol. Then, cells were subjected to centrifugation at 17,000 × g for 5 minutes, and the supernatant was decanted. The resulting pellet was mixed with 25 μL of 70% formic acid and 25 μL of acetonitrile, and then centrifuged for 5 minutes at 17,000 × g. A 1 μL aliquot of the resulting supernatant was immediately spotted onto a MSP 96 polished steel target plate (Bruker Daltonics; Billerica, MA, USA) in triplicate. After air drying for 15 minutes, 1 mL of CHCA matrix solution (CHCA in 50% acetonitrile/2.5% trifluoroacetic acid) was applied on top of each spot, and allowed to air dry for additional 15 minutes.

### Mass spectra acquisition

MALDI-TOF MS analyses were performed using a Bruker Microflex LRF MALDI-TOF mass spectrometer (Bruker Daltonics; Billerica, MA, USA) equipped with a nitrogen laser (337 nm) under the control of FlexControl software (version 3.0; Bruker Daltonics; Billerica, MA, USA). Mass spectra were manually collected in positive linear mode within a mass range from 2 to 20 kDa. Ion source voltages 1 and 2 were set at 20 and 18.15 kV, respectively. The lens voltage was set to 9.05 kV. Each spectrum was obtained by accumulation of 500 laser shots in 100 shot increments. Mass calibration was performed using a standard calibrant mixture including Adrenocorticotropic Hormone (ACTH)_clip(1-17) (human) (2093 Da), ACTH_clip (18-39) (human) (2464 Da), insulin oxidized B (bovine) (3495 Da), insulin (bovine) (5731 Da), cytochrome_C (equine) (12362 Da) and myoglobin (16952 Da).

### Data analysis

Raw mass spectra were exported as. txt files using FlexAnalysis software (version 3.0; Bruker Daltonics; Billerica, MA, USA) and imported into BioNumerics 7.1 software (Applied Maths, Sint-Martens-Latem, Belgium). The raw spectra were preprocessed using the default preprocessing templates in the BioNumerics 7.1 software, which include baseline subtraction using a rolling disc algorithm, continuous wavelet transformation noise estimation, and Kaiser window smoothing. Each peak with a signal to noise ratio of at least 10 was annotated.

All subsequent data analyses were conducted in BioNumerics 7.1 software (Applied Maths, Sint-Martens-Latem, Belgium). For each species, triplicate technical replicate spectra were summarized in a composite spectrum using a similarity filter of 95%. Curve-based cluster analysis including all replicates of spectra of pure cultures and the model mixture was performed by calculating pairwise Pearson product-moment correlation coefficients, and a dendrogram was constructed using the unweighted pair group method with arithmetic averages (UPGMA). Multidimensional scaling (MDS) was used to visualize further the similarity of the mass spectra. Peaks in spectra of pure cultures were matched to identify characteristic peaks for each bacterium using constant and linearly varying tolerance values of 2 m/z and 550 ppm, respectively^25^. A characteristic peak was manually selected for the species when this peak appeared in all of the three replicate spectra of the bacterium.

A synthetic mixture mass spectrum (SMS) was constructed by summarizing all of the 18 processed spectra of the pure cultures with a similarity filter of 0.5%. The intensity of each point in the synthetic spectrum was calculated by averaging the respective signal intensities in all the mass spectra. The similarity of the synthetic mixture spectrum was compared to the acquired mixture spectra (AMS) using the Pearson product-moment correlation coefficient.

### Identification of blind-coded samples

Blind-coded mixtures were constructed by mixing bacterial cell suspensions (O.D._600_ = 1) into sterile microcentrifuge tubes (Table 3). Mass spectra of the blind-coded mixtures were acquired and pre-processed as described above. Potential biomarkers for each species were identified based on peak matching results and only peaks with intensities higher than 500 a.u. were considered. Synthetic mixture spectra were constructed by summarizing spectra of pure cultures. Species in blind-coded mixture samples were identified by comparing the acquired mixture spectra of the blind-coded samples to the synthetic mixture spectra (similarity coefficient-based) and by identifying species-specific peaks (potential biomarkers) in the acquired mixture spectra (biomarker-based).

## Additional Information

**How to cite this article**: Zhang, L. *et al.* Biomarker- and similarity coefficient-based approaches to bacterial mixture characterization using matrix-assisted laser desorption ionization time-of-flight mass spectrometry (MALDI-TOF MS). *Sci. Rep.* **5**, 15834; doi: 10.1038/srep15834 (2015).

## Supplementary Material

Supplementary Information

## Figures and Tables

**Table 1:** Quality and reproducibility of mass spectra of individual bacteria and the model mixture.

ID	Bacteria^a^	Gram	Reproducibility^b^ (%)	Number of peaks^c^	Mass range (Da)^c^	
					Lowest mass	Highest mass
F8	*Moraxella* spp.	-	96.0 ± 2.4	63 ± 6	2141	10002
F14	*Exiguobacterium* spp.	+	99.7 ± 0.2	29 ± 3	2134	11822
M14	*Kocuria* spp.	+	96.7 ± 2.5	38 ± 6	2188	9646
M15	*Brevibacterium* spp.	+	98.3 ± 1.3	60 ± 2	2067	9909
R4	*Aminobacter* spp.	-	98.6 ± 0.7	36 ± 3	2231	9639
R8	*Curvibacter* spp.	-	98.8 ± 0.9	39 ± 1	2026	10846
Mix	Mixture		99.6 ± 0.2	135 ± 10	2027	11825

^a^Bacteria were isolated from Kartchner Caverns, AZ, USA and identified using 16S rDNA sequencing^25^.

^b^Values reported are the average correlation coefficients of 3 technical replicates ± one standard deviation.

^c^Values reported are the average values of 3 technical replicates ± one standard deviation.

**Table 2:** Comparison of peaks in mass spectra of bacterial isolates and the mass spectrum of the model mixture.

ID	Number of peaks observed in the mixture spectrum (N_m_)	Percentage of presence (PP)^a^
F8	26	41.3
F14	22	75.9
M14	13	34.2
M15	14	23.3
R4	20	55.6
R8	35	89.7

^a^PP = [Number of peaks observed in the mixture spectrum (N_m_)/Number of peaks observed in the spectra of the pure culture (N_p_)] * 100.

**Table 3:** Identification of blind-coded mixture samples based on comparison of the similarity coefficients between acquired spectra and synthetic spectra.

ID	Composition	Smoothing (%)	Similarity coefficient (%)^a^	Multiple identification results (Yes/No)^b^	Species identified
A	F8, R4	0	90.0	No	R8, R4
B	F14, R8	0	92.9	No	F14, R8
C	F14, M15	−c	−	−	−
D	M14, M15, R8	0.5	69.8	No	M14, M15, R8
E	F8, F14, M14, M15	1	74.4	Yes	F8, F14, M14, M15
			71.7		F8, F14, M14, M15, R4
			69.9		F8, F14, M14, M15, R4, R8
F	F8, F14, M14, M15, R4	0.5	71.1	Yes	F8, F14, M14, M15, R4, R8
			74.3		F8, F14, M14, M15
			75.1		F8, F14, M14, M15, R4
G	F8, F14, M14, M15, R4, R8	0.5	74.5	No	F8, F14, M14, M15, R4, R8

^a^Similarity coefficient was calculated using the Pearson correlation coefficient with various levels of smoothing (0–1%).

^b^Identification results were reported when similarity coefficients reached 68.6%.

^c^Similarity coefficient did not reach 68.6% even with 1% smoothing.

**Table 4:** Identification of blind-coded mixture samples based on potential biomarker peaks.

ID	Composition	Number of Biomarkers found for each species^a^	Species identified initially	Species identified after optimization
A	F8, R4	F8(11); M14(1); R4(6)	F8, M14, R4,	F8, R4
B	F14, R8	F14(8); R8(8)	F14, R8	F14, R8
C	F14, M15	F14(6); M15(2); R8(1)	F14, M15, R8	F14, M15
D	M14, M15, R8	F8(1); F14(2); M14(5); R8(8);	F8, F14, R8, M14	F14, R8, M14
E	F8, F14, M14, M15	F8(12); F14(6); M14(5); M15(9);	F8, F14, M14, M15	F8, F14, M14, M15
F	F8, F14, M14, M15, R4	F8(13); F14(8); M14(5); M15(6); R4(6); R8(2);	F8, F14, M14, M15, R4, R8	F8, F14, M14, M15, R4, R8
G	F8, F14, M14, M15. R4, R8	F8(11); F14(8 M14(6);); M15(6); R4(5); R8(8)	F8, F14, M14, M15. R4, R8	F8, F14, M14, M15. R4, R8

^a^Values in parentheses are the number of potential biomarker peaks found for each species in the blind-coded samples.
