# Supplementary material for: Biomarker- and similarity coefficient-based approaches to bacterial mixture characterization using matrix-assisted laser desorption ionization time-of-flight mass spectrometry (MALDI-TOF MS)
Source: Sci Rep. 2015 Nov 5;5:15834. doi: 10.1038/srep15834 (PMC4633581; doi:10.1038/srep15834)
Supplement: Supplementary Information [file srep15834-s1.doc]

Biomarker- and similarity coefficient-based approaches to bacterial mixture characterization using matrix-assisted laser desorption ionization time-of-flight mass spectrometry

(MALDI-TOF MS)

Lin Zhang, Sonja Smart and Todd R Sandrin*

School of Mathematical and Natural Sciences

Arizona State University

Phoenix, AZ 85069

**Supplementary Materials**

*Corresponding author

Todd R. Sandrin

School of Mathematical and Natural Sciences

MC 2352, PO Box 37100

Arizona State University

Phoenix, AZ 85069

E-mail: Todd.Sandrin@asu.edu

Phone: 602-543-6934

**Supplementary Table 1** Peaks observed in the mass spectrum of the model mixture for each isolate.

| **ID** | **Peaks (Da) observed in the mass spectrum of community** |
| --- | --- |
| F8 | 2140 , 2268, 2437, 2721, 2832, 3007, 3389, 3623, 4730, 5912, 5963, 5981, 6200, 6673, 6704, 6743, 6858, 6986, 7017, 7430, 7565, 7710, 8722, 8755, 9511, 9556 |
| F14 | 2140, 2590, 2796, 3048, 3412, 3685, 4247, 4587, 4730, 5105, 5145, 6063, 6564, 6792, 6838, 7340, 7738, 9153, 9440, 10192, 11824 |
| M14 | 2188, 2868, 3074, 3215, 4343, 4618, 5105, 5849, 6116, 6143, 6397, 7380, 8532 |
| M15 | 2180, 2197, 2208, 3007, 3215, 3444, 3730, 4327, 4685, 6397, 6435, 7430, 8680, 9511, |
| R4 | 2437, 3105, 3352, 3585, 3709, 4247, 4655, 4840, 5632, 6178, 6673, 6743, 6767, 6986, 7141, 7390, 9153, 9179, 9288 |
| R8 | 2026, 2041, 2084, 2098, 2112, 2167, 2268, 2281, 2324, 2339, 2473, 2570, 2868, 3143, 3264, 3444, 3570, 3709, 3904, 4301, 4673, 5105, 5208, 5429, 6253, 6496, 6858, 7109, 7380, 7781, 9326, 10192, 10403, 10847 |

^a^ Peaks had a minimum intensity of 100 a.u. and were observed in all triplicate spectra.

**Supplementary Table 2** Peaks observed in the mass spectra of the model mixture but not assigned to any component bacterium.

| Peak (m/z) | Intensity (a.u.)^a^ | Number of replicate spectra containing the peak^b^ |
| --- | --- | --- |
| 2121 | 460 ± 144 | 0 |
| 2415 | 464 ± 33 | 2 (R4) |
| 2906 | 143 ± 4 | 0 |
| 3883 | 265 ± 25 | 1 (F14) |
| 4478 | 239 ± 28 | 2 (F14) |
| 4978 | 261 ± 24 | 1 (F8); 1 (F14) |
| 6022 | 450 ± 46 | 0 |
| 6532 | 186 ± 63 | 1 (M15) |
| 6544 | 209 ± 71 | 2 (R8) |
| 6620 | 176 ± 2 | 1 (R4) |
| 6897 | 751 ± 19 | 0 |
| 8934 | 214 ± 14 | 1 (F14) |
| 9350 | 187 ± 9 | 0 |

^a^ Intensity values are the means of triplicate spectra ± one standard deviation.

^b^ Bacterial IDs are specified in the parentheses.
